# Miniature optical fiber curvature sensor via integration with GaN optoelectronics

**DOI:** 10.1038/s44172-022-00049-w

**Published:** 2022-12-27

**Authors:** Fan Shi, Hao Zhang, Ziqi Ye, Xianwu Tang, Feifei Qin, Jiabin Yan, Xumin Gao, Hongbo Zhu, Yongjin Wang, Yuhuai Liu, Hiroshi Amano

**Affiliations:** 1 GaN Optoelectronic Integration International Cooperation Joint Laboratory of Jiangsu Province, Nanjing University of Posts and Telecommunications, Nanjing, 210003 China; 2 National Center for International Joint Research of Electronic Materials and Systems School of Information Engineering Zhengzhou University, Zhengzhou, 450001 China; 3 Institute of Materials and Systems for Sustainability Nagoya University, Nagoya, 464-8601 Japan

**Keywords:** Optics and photonics, Applied optics

## Abstract

Optical fiber curvature sensors have been considered as a promising option for human motion detection due to its good toughness, bending flexibility and anti-electromagnetic interference. However, for wearable devices, the miniature configuration is preferred, and a high integration of the light emitter, receiver and guided fiber is essential to configure the miniaturized sensing system. Here, we present a miniaturized curvature sensing system by integrating a GaN-based optoelectronic chip with the plastical optical fiber (POF). The light emitter and detector are fabricated on a GaN-on-sapphire wafer to form a tiny chip sized at 2.5 $\times$ 1.5 mm^2^. The on-chip photodetector (PD) effectively senses the reflected light intensity, extracting information on the fiber bending deformation. A compact curvature sensing system is demonstrated for finger motion detection with movement angles of 30–90° and frequencies of 0.4, 1, and 1.6 Hz. The results show that the monolithically integrated LED and PD chip can be combined with the POF with reliable operation. The demonstration of the monolithically integrated optoelectronic device suggests a promising potential technology for future wearable fiber optical sensor system.

## Abstract (web summary)

Shi and colleagues create a miniature curvature sensor by integrating a light emitting diode and GaN-based photodetector into a plastic optical fiber. They detect finger motion to show potential for wearable sensing systems.

## Introduction

Lightweight, low-cost, and miniaturized curvature sensing systems are highly desirable for human motion detection, such as respiration monitoring, joint angle assessment, and exercise frequency measurements^1–3^. In particular, optical fiber-based curvature sensors have recently attracted attention because of their low cost, resistance to corrosion, and anti-electromagnetic interference^4,5^. In principle, transforming the strain or bending deformation into the intensity or phase changes the optical signals traveling along the fiber. Thus, the condition of the target object can be monitored. To date, a variety of all-fiber sensing structures have been developed, such as multimode interference structures^6,7^, long-period fiber grating^8,9^, D-shaped fiber structures^10,11^, fiber Brag grating^12,13^, and others. Although some silica fiber sensor systems have achieved high sensitivities, the sensing range is limited in the low curvature region (<5 m^−1^) due to the rigidity of silica fibers^14^. Moreover, their optical configurations are generally bulky and costly because of the expensive laser source and complicated optical interference path.

Compared to silica fiber, plastic optical fibers (POF) have the merits of a larger core size and higher numerical aperture^15,16^, which allows light emitting diodes (LEDs) to be an economical alternative to laser sources. Besides, POFs have better flexibility and can be employed in scenarios with a considerable level of bending deformation. A POF sensing system typically requires an LED as a light source, a segment of the fiber as a sensing unit, and a photodetector (PD) as the detection part. With this traditional layout, POF sensors have been well proven in a series of applications, such as hand gesturing, joint activity, and strain measurement.

Research on miniaturized POF sensing systems has increased greatly as driven by wearable application scenarios^17–19^. A portion of them is based on a modular design concept, which integrates the light source, sensing unit, and detector in space to form a compact sensing architecture. For example, Wang et al.^19^ proposed a low-cost, wearable, textile-based, respiratory sensing system. In the approach, a D-shaped POF sensor was fixed on an elastic belt to sense bending deformation, and an LED light source and optical receiver were customized with small packaging. Di et al.^20^ developed a hand gesture monitoring system based on fiber-optic curvature sensors. Similarly, modular designs of the light emitter and receiver are still applied to reduce the package size.

Another candidate is based on the integration of a smartphone with the POF, where the pixel intensity change from the camera is monitored for sensing^21–23^. Based on smartphone hardware (flashlight, camera, etc.) as a light source and detector, Aitkulov et al.^21^ presented an all-POF smartphone setup capable of detecting breathing rates through intensity-based modulation. However, existing demonstrations have a common issue where the light source and detector are still discrete, resulting in a large space occupation and test instability, which highly limits the scope of its practical application. Improving the integration of the sensing system architecture has become a concern of researchers. Toward this goal, removing the discrete LED source and PD is a critical step to miniaturize the POF sensing system.

Benefiting from the rapid development of third-generation semiconductor materials, GaN-based LEDs have become the mainstream illumination sources due to their long lifespan, high efficiency, and high environmental stability. Besides illumination, GaN semiconductors provide a promising platform to construct chip-level integrated devices. The integration of other optical components, such as detectors and waveguides, on a single wafer has been well proven^24–26^. As InGaN/GaN multi-quantum well (MQW) structures have the dual functions of optical emission and reception^27–29^, this opens a window in the fields of duplex optical communications, reverse optical modulations, and optical sensing.

This paper presents a proof of concept to integrate an optoelectronic chip with the POF to further implement a reflective sensing architecture between the chip and fiber through a fiber end-mounted mirror. The chip is composed of an LED and PD grown on a single GaN-based material platform through micro-nano processing technology. A monolithic integration design is used to replace the external discrete light-emitting and detection elements with a tiny chip, and a reflective-type optical interconnect is built to further miniaturize the sensing system. The proposed POF sensor exhibits a large curvature measurement with a detection range from 0–8.43 m^−1^, which makes it suitable for wearable sensing scenarios with human motion. The results provide ideas to develop the next generation of optoelectronic systems on fiber substrates.

## Results

**Fig. 1:**
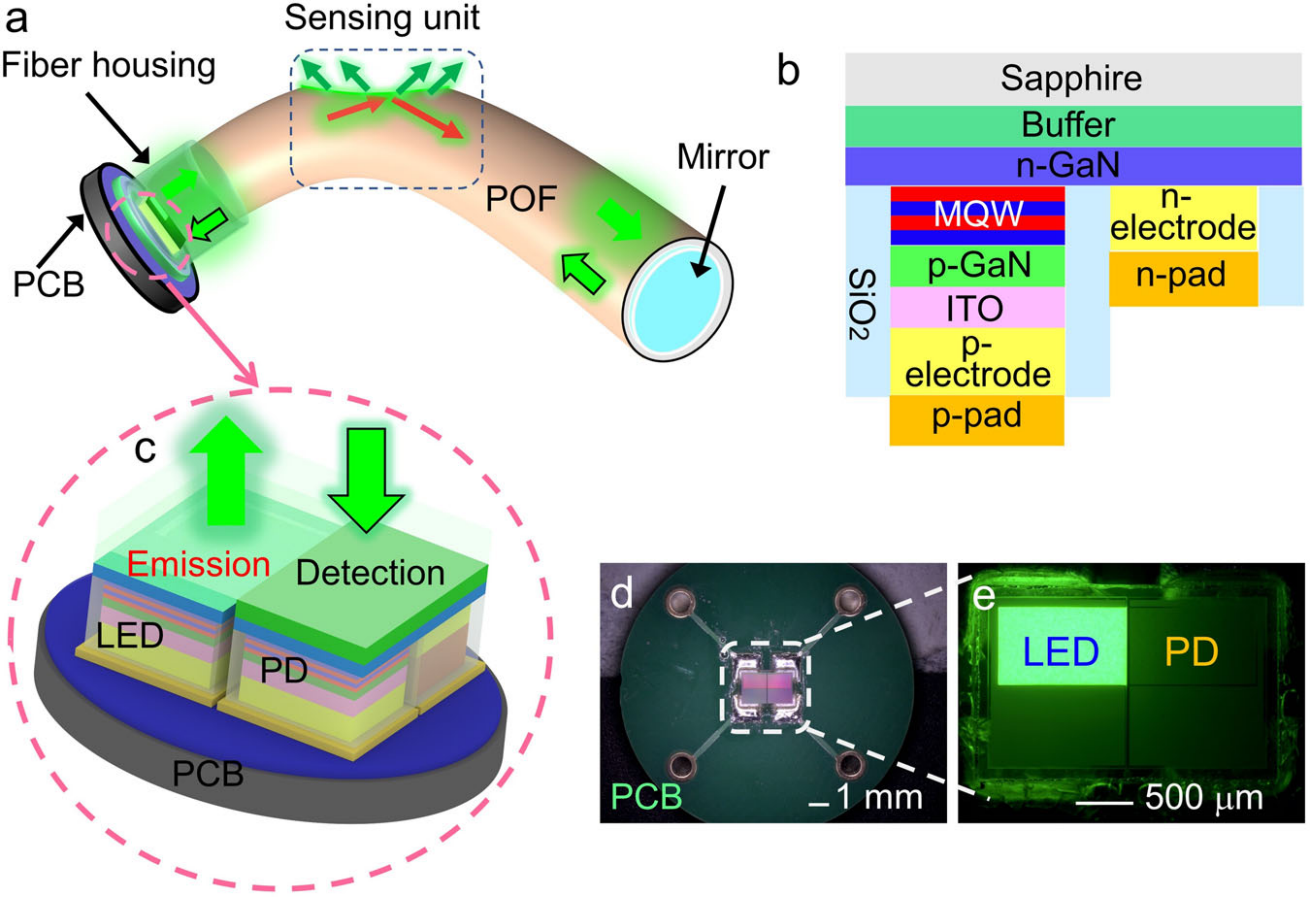
Fabrication processes of the wearable sensor. **a** Schematic of the integration of GaN-based optoelectronic chip and POF (POF, plastic optical fiber). **b** Cross-sectional view of the same structure of the LED (LED, light-emitting diode) and PD (PD, photodiode). **c** Emission-detection monolithic integrated chip with flip-chip structure. **d** Photograph of the LED-PD chip bonded to a PCB (PCB, printed circuit board) with a diameter of 1.45 cm. **e** Magnified image of the chip operating at a bias voltage of 2.4 V.

A schematic of the reflective POF sensing system using a relatively small optoelectronic chip is shown in Fig. 1a. Within the structural layout, the POF with a D-shaped region is adopted as the sensing unit, and the monolithically integrated LED-PD chip with the same MQW structure acts as the light emitter and receiver.

The monolithically integrated LED-PD chip is the key to eliminate the traditional discrete optics layout and miniaturize the sensor system architecture. The light emitter and receiver with the same MQW structure are schematically depicted in Fig. 1b. A magnified image of the chip layout is presented in Fig. 1c. The flip-chip structure of the device is designed to enhance the luminous flux and simplify the coupling to the POF. Figure 1d shows an optical image of the fabricated chip mounted on a printed circuit board (PCB). A magnified image of the LED-PD chip sized at 1.5 $\times$ 2.5 mm^2^ is shown in Fig. 1e, which operates at a bias voltage of 2.4 V.

### Light emission and detection of the monolithically integrated chip

**Fig. 2:**
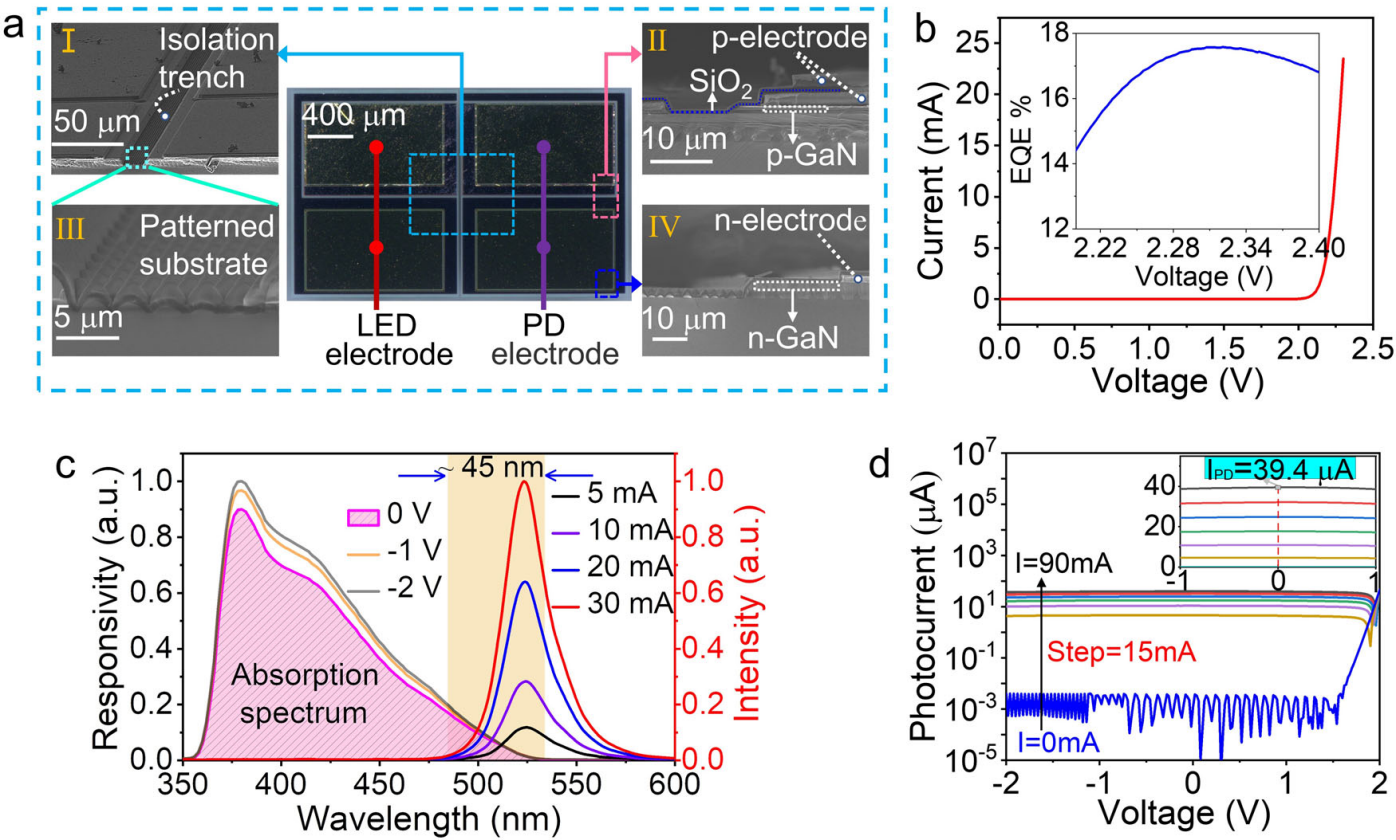
Characteristics of the GaN optoelectronic chip. **a** The microscope image and SEM images of the fabricated chip, (I) SEM image of the center of chip, (II) SEM image of the cross-section of PD anode, (III) SEM image of the patterned substrate of isolation trench, (IV) SEM image of the cross-section of PD cathode. **b** Current-voltage (*I*–*V*) characteristics of the LED, where the inset shows the external quantum efficiency (EQE) of the LED. **c** Electroluminescence (EL) spectrum of the on-chip LED and absorption spectra of the on-chip PD biased at 0 V, −1 V, and −2 V. **d** *I*–*V* characteristics of the PD measured with (injection current changing from 15 mA to 90 mA with a step of 15 mA) and without (blue curve) LED irradiation. The inset shows *I*–*V* curves in the linear coordinates.

Figure 2a shows the microscope image of the fabricated chip, in which the LED and PD is monolithically integrated on a wafer. As marked with the light blue dotted box, a trench exists between the LED and PD for device isolation. The typical patterned substrate indicates the isolation trench is etched to the sapphire substrate. The pink dotted box marked shows the cross-section structure of PD anode, in which the deposited SiO_2_ layer, the p-mesa and electrode can be clearly seen. The dark blue dotted box marked region represents the edge of PD cathode, where the interface between the n-mesa and patterned substrate can be seen.

Effective detection of the signal transmitted in the fiber channel depends on stable illumination from the LED source and high isolation between the on-chip LED and PD. The current-voltage (*I*–*V*) curve of the LED is measured using a semiconductor device analyzer (Agilent B1500A). As plotted in Fig. 2b, the forward-biased voltage is at 2.2 V, and a steep I-V curve indicates the well-deposited Ohmic contact. The inset shows the green light emission has a maximum external quantum efficiency (EQE) of 17.5%. The photoelectric efficiency of the GaN optoelectronic chip is measured to be 2.64% under 1-sun illumination (Supplementary Fig. s2). As the same MQW structure is used both for light emission and detection, it is necessary to verify the ability of a monolithically integrated PD to sense the LED emission. The electroluminescence (EL) spectrum of the LED and the absorption spectrum of the PD are tested and plotted in Fig. 2c. The EL spectrum of the green LED is measured using an optical spectrometer (Ocean Optics USB4000), where the radiation wavelength is around 525 nm. The response spectrum (RS) of the PD is obtained by the monochromatic light irradiation as filtered from a broadband light source. There is a partially overlapped wavelength region between the RS and EL spectra, which proves that the same MQW structure can be used as either a light emission device or a detection device. This emission-detection overlapping phenomenon results from the Stokes shift between the absorption and emission energies in the InGaN layer. As the LED injection current increases (from 5 to 30 mA), the peak wavelength of the corresponding EL spectrum gradually shifts to shorter wavelengths, which broadens the overlapping region. That is, the monolithically integrated PD can more easily sense the LED emission with a higher driving current. Compared with enhancing the LED emission, applying a reverse bias (such as −1 or −2 V) on the PD side has a weak effect on the bandwidth of the overlapped region. Through the Stokes shift effect, the RS exhibits a decreased absorption trend at longer wavelengths, which gives the PD a lower detection efficiency in the overlapped region. To verify the performance of the PD, *I*–*V* curves for the PD under illumination from the on-chip LED are measured as shown in Fig. 2d. The dark current is in the low current level of the order of 10^−10^ to 10^−9 ^A due to the device isolation. As the driven current of the LED is turned from 0-90 mA with a step of 15 mA, the photocurrent generated by the PD gradually increases and reaches 39.4 μA at 0 V, as depicted in the inset of Fig. 2d, which implies a good detection performace of the PD.

### Chip and POF integration sensing system

**Fig. 3:**
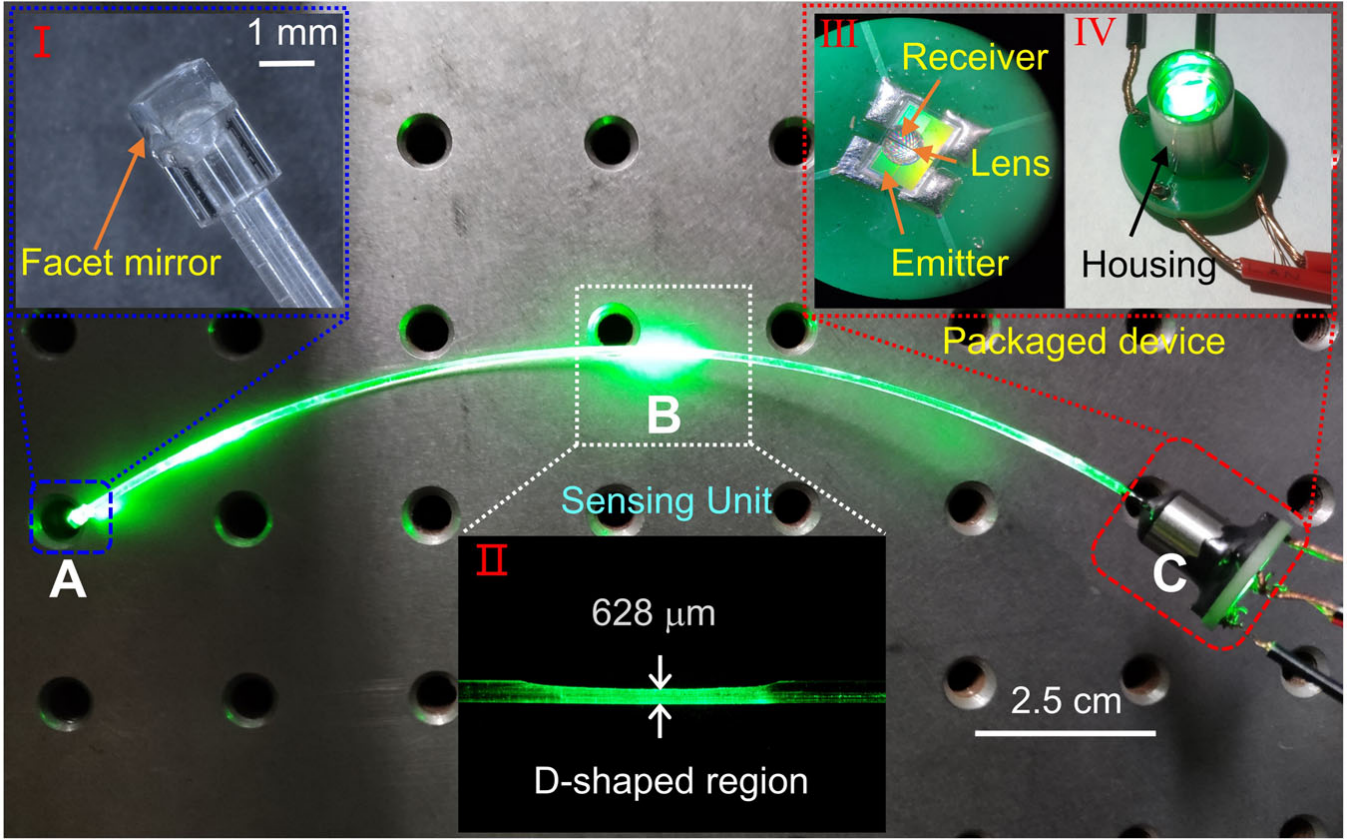
Bird’s-eye optical image of the chip-integrated POF wearable sensing system. “A” part is the reflection end, forming a coaxial reflection in the fiber link. The inset image “I” shows a mirror attached to the fiber end facet. “B” part is the sensing unit, and the inset image “II” shows a D-shaped structure fabricated on the POF with a diameter of 628 μm. “C” part includes the elements of emitter and receiver, where a lens formed by high refractive glue is deposited on the emitter and receiver for beam convergence (III), and the encapsulation of optoelectronic chip and POF is implemented by a housing (IV).

The properties of the emission-detection of the on-chip devices indicate the fabricated chip can integrate with the POF for miniaturized fiber systems. An optical image of the built fiber sensing system based on the monolithically integrated chip is displayed in Fig. 3. A detailed fabrication process is provided in Supplementary Fig. s3. Part “A” is the reflection unit, as marked with a blue dotted box, where a mirror is coated on the fiber end facet to form a fiber reflector (can be seen in the inset I). Part “B” is a piece of 15-cm long POF with a 1-cm long D-shaped sensing unit. As depicted in the white dotted box, the fiber diameter is reduced to 628 μm after side polishing (can be seen in the inset II). The POF used in the experiments has an original 980 μm diameter core and 10 μm cladding thickness. Reducing the fiber diameter helps enhance the sensing ability. Part “C” is the light-emitting and detection unit, which is packaged in a ~1.5 cm long steel tube. As illustrated in the red dotted box, the chip is first fixed on the center of the PCB and then a drop of high refractive glue is deposited on the sapphire surface to form a micro hemispherical lens (can be seen in the inset III). This converges the light and enhances coupling to the POF facet. Then, the mounted chip and POF are packaged using a steel housing (can be seen in the inset IV). With this design, a light emitter and receiver are assigned at one side, and the sensing probe is at the other side, which leads to a compact and reflected lightpath layout. Changes in the external environment are converted into the deformation of the D-shaped POF, and the reflected light carried with external modulation information is transmitted through the fiber channel, before finally being received by the on-chip PD.

**Fig. 4:**
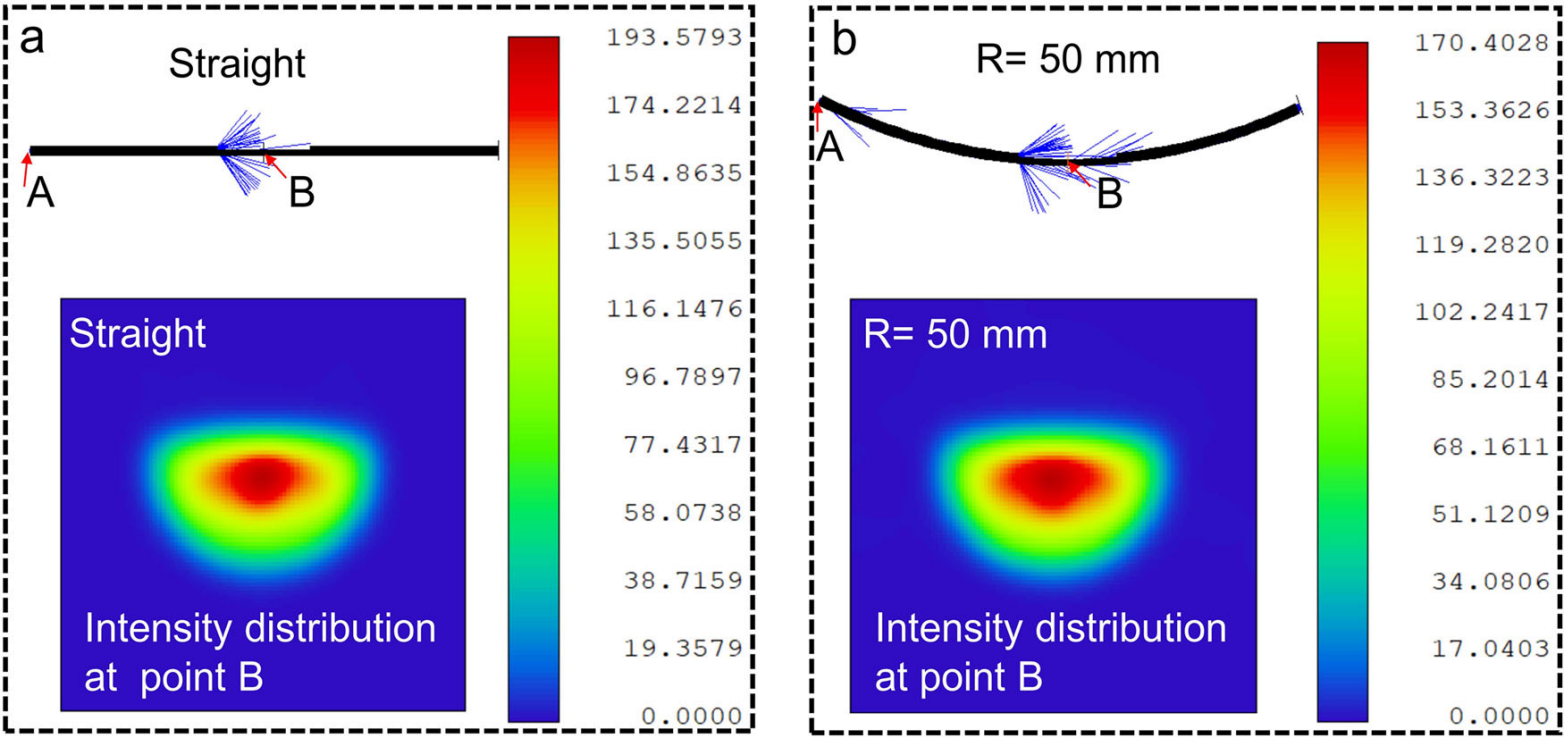
Ray tracing and light intensity distribution for the POF system without/with bending. Simulation results of POF in a (**a**) straight state and (**b**) a bending state with 50 mm radius of curvature, where point A and B represent the positions of light emitter and monitor, respectively.

The operating mechanism of the curvature sensing system is based on the attenuated total internal reflection via bending. Light propagation through the POF is studided by ray-tracing analysis. Figure 4a, b show the depicted 3D models and light propagation behavior of the POF with the same fiber length and diameter under straight and bending states, respectively. As illustrated in the ray tracing, partial light escapes from the D-shaped region and propagates in the air in the form of evanescent waves. Fiber deformation will affect the strength of the evanescent field, so a specific relationship is established between the amount of light intensity change and the amount of fiber deformation. Under the same amount of rays emitted from the light source (point A), the density of rays remaining in the D-shaped region (point B) reduces when the fiber is bent, leading to the weak received light. For comparison, a bending radius of 50 mm is simulated, and the obtained maximum intensity value at the D-shaped region reduces from 193.5 to 170, as presented in the intensity color bar.

**Fig. 5:**
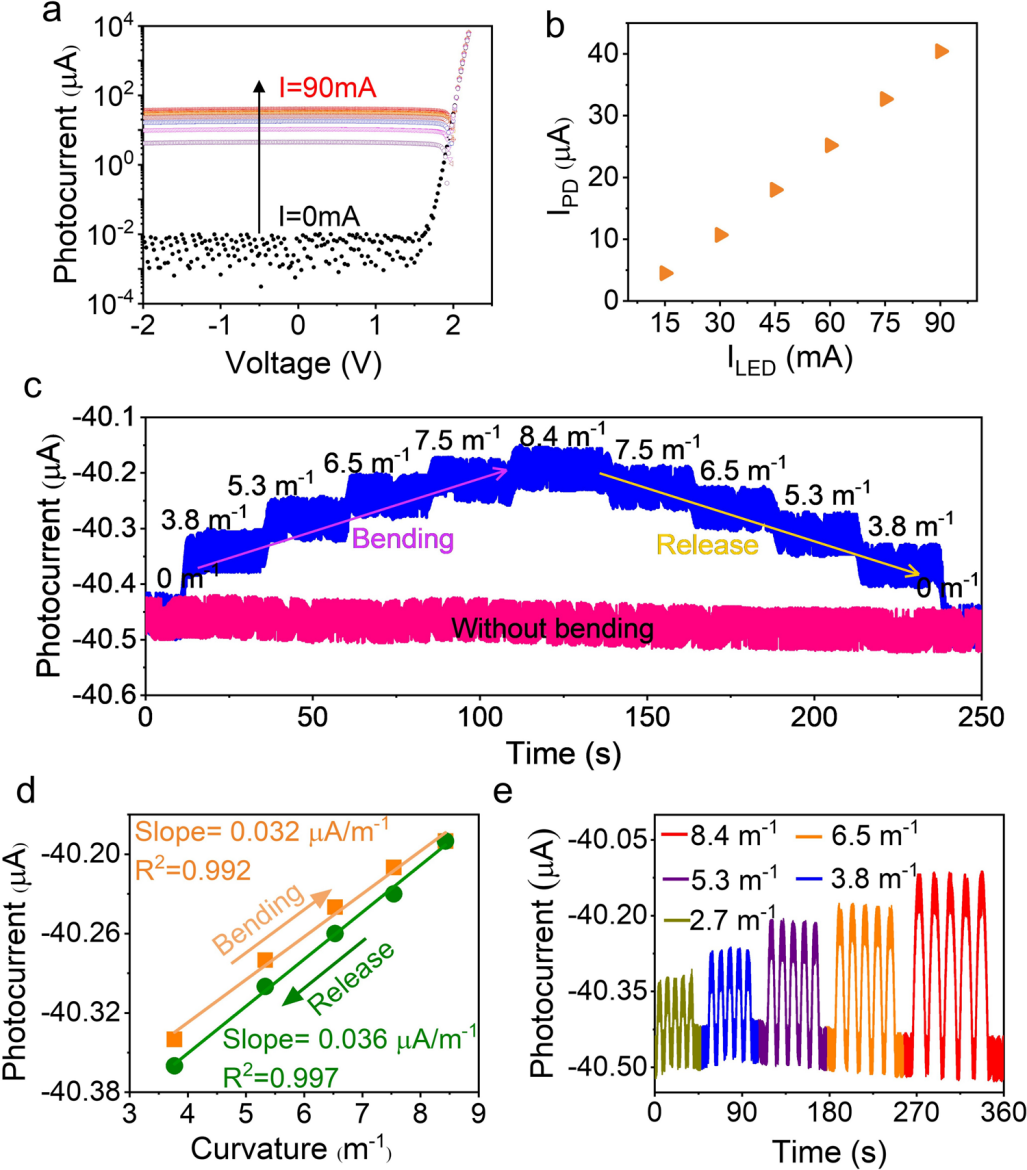
Curvature tests of the chip-POF integrated system. **a** *I*–*V* curves of the detector, where the black dotted line represents the photodetection of the PD without light irradiation, and the colored dotted lines display the generated photocurrent under the on-chip LED operating at currents ranging from 15–90 mA. **b** Generated PD photocurrents (I_PD_) versus different LED driving currents (I_LED_). **c** Photocurrent response of the sensing system as measured under different fiber bending conditions (without bending plotted as pink baseline and continuous bending depicted as the step-like blue curve). **d** Measured photocurrent versus curvature, where the solid lines are the linear fit to the data. **e** Real-time photocurrent response to instantaneous curvature changes.

Once the LED-PD chip has been integrated on the POF, measurements are conducted to quantify the system performance by monitoring the photocurrent of the on-chip PD. First, the *I*–*V* curves of the PD under different LED injection currents are characterized to check the optoelectronic characteristics of the device after encapsulation. As shown in Fig. 5a, a series of *I*–*V* curves for the on-chip PD are swept when the injected LED current changes from 0-90 mA. Without illumination, the photocurrent is between the level of 10^−9^ and 10^−8 ^A, which is consistent with the data in Fig. 2d. Once the LED is turned on, the RS of the PD and EL of the LED are partially overlapped, and photocurrent generated by the PD increases with the injected current. The relationship between the photocurrent I_PD_ and injected current I_LED_ is plotted in Fig. 5b, which shows good linearity.

Compared to the photocurrent response of the bare chip in Fig. 2d, the end face reflection enhances the photocurrent when the chip is integrated with POF. For example, the photocurrent produced by the PD increases from 39.4 μA to 40.5 μA at the same 90 mA injection current. Obviously, the varitity of photocurrent response is contributed by the reflected light from the fiber end-mounted mirror. A bending sensing test setup was built to quantitatively characterize the bending response of the proposed sensing system, which is often utilized in curvature measurements.

The moving stage is driven by an external motor and moved continuously with a speed of 2.3 mm/s. The curvature response of the system is then studied, and changes in the photocurrent are recorded with a fixed injection current of 90 mA. A continuous increase and decrease of the curvature from 0–8.43 m^−1^ is loaded on the fiber by moving the stage. The holding time between the curvature change is around 20 s. The measured results presented in Fig. 5c show a step-like response curve (blue curve) that resembles a staircase. The difference in photocurrent reaches ~250 nA when the curvature changes from 0–8.43 m^−1^. Figure 5d shows the relationship between the photocurrent and curvature, and the measured data is linearly fitted. For the step-up and step-down processes, the sensitivities are estimated to be around 32 and 36 nA/m^−1^, respectively. The slight difference may be from the inherent current jitter, as shown in the plotted pink baseline in Fig. 5c. As illustrated in Fig. 5d, the fiber system displays a high reproducibility under different curvature changes. The dynamic photocurrent response is consistent with the data in Fig. 5c. The long-term durability of the sensing system is tested within 1.8 h by monitorig the photocurrent difference jitter (Supplementary Fig. s4). The results indicate that the system is relatively stable and reproducible under repeated bending and release cycle test.

### Finger motion detection

The advantages of large curvature range and bending flexibility promotes the application of POF in wearabale sensing. In fact, the maximum curvature radius of the POF with D-shaped structure can reach ~15 mm, while it can also return to the original state after the bending release. Besides, it has been reported that the maximum bending angle can also reach 200°^16^. To demonstrate the utility of our proposed system in wearable perception scenarios, we fixed it on the fabric of gloves to test its capacity for finger motion detection. For the finger joint movement detection, several factors should be considered, e. g., undesired fiber bending due to the loose fixtures and errors on the sensor response caused by the hysteresis of POF. To restrict the undesired fiber bending, reliable mechanical positioners for the fiber are preferred. To reduce the hysteresis of the POF, decrease of the fiber length is a straightforward and effective method^16,17^. In our tests, a piece of relatively short POF (~15 cm) is adopted, which helps reduce the hysteresis and increase the sensor repeatability. Besides, the sensing zone on the POF is firmly fixed and attached to the finger joint to reduce the measurement error resulted from the undesired bending.

**Fig. 6:**
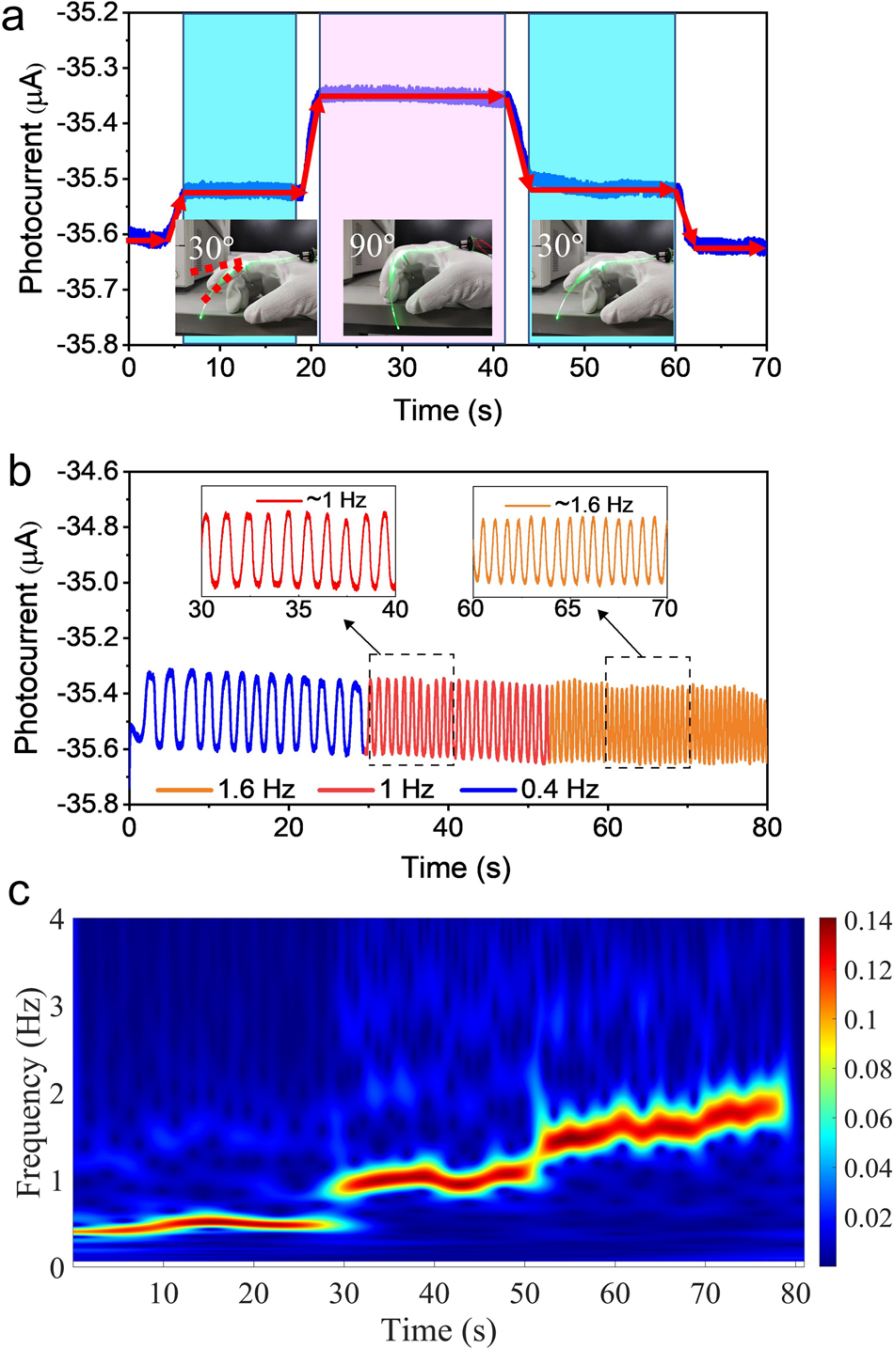
Finger joints motion detection. **a** Photocurrent response under different finger joint angles. **b** Different bending frequencies under 90° bending angles. **c** Time-frequency distributions of the bending frequency signal change from slow to fast.

Figure 6a shows the photocurrent response under a large bending scale of finger joint angles from 30–90°. Herein, the injected LED current is ~80 mA, and the corresponding baseline of the photocurrent is around −35.6 μA. The current gradually decreases from the baseline value as the finger bend angle increases. A current step is formed when the finger is held in a constant bent state (Supplementary Movie 1). The bending and releasing process of the finger produces a nearly symmetric photocurrent magnitude distribution, which indicates the high stability of the wearable device. The real-time photocurrent response curve is an important measurement to further verify the detection performance of the finger bending frequency. The response of the fiber sensor when the finger is repeatedly bent with different motion frequencies is illustrated in Fig. 6b (Supplementary Movie 2). The photocurrent response curve contains the frequency and amplitude information for the finger motion. The amplitude corresponds to the bending angle, which is consistent with the data in Fig. 6a. The angle for finger bending is around 90°. A straightforward approach for the frequency information is to count the number of waveform cycles. The insets of Fig. 6b give an approximate estimate of 0.4, 1, and 1.6 Hz. Normally, precisely analyzing the frequency domain characteristics of a signal requires the use of Fourier transformations. However, this is used primarily to process stationary signals. Through the Fourier transform, the frequency components contained in the overall signal can be obtained, but the time when each component appears cannot be discerned. For non-stationary signals, besides the frequency information, it is also necessary to know the time when each frequency appears, which is called time-frequency analysis. Short-time Fourier transform (STFT) based on wavelet transformation provides a good solution in which the frequency can be obtained along with the appropriate time. Figure 6b shows the time-domain waveforms for pulses during finger joint motion and changes in the frequency from slow to fast, respectively. After the STFT analysis, the time-frequency distribution of the finger motion is presented in Fig. 6c. The participant’s finger motion from 0–30 s is from 0.4–0.5 Hz, 30–52 s is from 0.9–1 Hz; and 52–80 s is from 1.43–1.85 Hz. The STFT technique allows displaying the instantaneous information of the finger motion, which helps extract transient information for gesture control and recognition.

## Discussion

We propose and demonstrate an effective method to construct a portable POF-based sensing system. A monolithically integrated optoelectronic chip is packaged on the fiber facet to replace the bulky external light source and detector to form a small footprint and low-cost structural layout. On the one hand, the same MQW structure fabricated on a single GaN platform provides dual functions as an LED light source and PD, which promotes mass production and process compatibility. On the other hand, the monolithic integration of the LED and PD utilizes an optical reflected sensing layout, which provides a flexible probe-style sensor head compared to the transmissive configuration. To demonstrate the effectiveness of our technique, the curvature characteristics of the POF sensor are determined, which shows a large curvature detection from 0–8.43 m^−1^ and exhibits a good linearity and repeatability. The large curvature range and fast response allow applications in human joint motion monitoring, such as finger motion scenarios (different bending angles and frequencies) given in our experiments. These proof-of-concept experiments validate the effectiveness of the technique to integrate optoelectronic chips on fiber, enabling fascinating application prospects for miniaturized sensing systems in wearable devices.

## Methods

### Fabrication of the LED-PD chip

The chip is designed and fabricated through micro-nano processing technology on 4-inch commercial GaN-on-sapphire LED epitaxial wafers. The epitaxial layers on the sapphire substrate include an AlN buffer layer, an unintentional-doped GaN layer, an N-doped GaN layer, an InGaN/GaN MQW layer, and a p-GaN layer from the bottom to the top. The fabrication step of the photonic chip is similar to our previous work^30^. First, a transparent indium tin oxide (ITO) current spreading layer is deposited via sputtering. After rapid thermal annealing treatment in an N_2_ atmosphere for 7 min, two 1.2 $\times$ 0.72 mm^2^ rectangular mesa regions are photolithography defined as the LED and PD regions. The n-GaN surface is exposed using inductively coupled plasma reactive ion etching (ICP-RIE) with an etching depth of 1.4 μm. A 20-μm-wide trench is formed from a deeper ICP etch for device isolation. Subsequently, an SiO_2_ passivation layer is coated via plasma-enhanced chemical vapor deposition (PECVD), and apertures are opened on it. Finally, the Ti/Au metals are sputtered by magnetron sputtering to form the n-pad and p-pad. A detailed description of the procedure is given in Supplementary Fig. S1.

### Curvature measurements

Two ends of the chip-POF integration fiber system are fixed on the clampers, respectively. One translation stage remains stationary, and the other moves inward to induce bending. As the fiber is bent inward, it is usually approximated as an arc circle. The bending curvature (*C*) of the sensor is defined as: $C=2h/({h}^{2}+{L}^{2})$, where *h* is the bending displacement at the center of D-shaped fiber, *L* is the half of distance between two clampers.

### Simulation methods

Light propagation behavior along the POF is simulated by the commercial software Zemax. Firstly, the designed POF models with straight and bending states are pre-drawn by Computer Aided Design (CAD) software. The POF model has the core/cladding thickness of 980/10 μm, respectively. The D-shaped region has a uniform length of 1 cm with a thickness of 620 μm. Secondly, the designed D-shaped POF from the CAD software is uploaded into Zemax software. The refractive index of fiber core and cladding are set to 1.49 and 1.41, respectively. The wavelength of the light source is set to 530 nm, and the maximum angle is defined as 60°. The detector named “Detector Rectangle” is used to monitor the intensity distribution of the fiber cross-section in the D-shaped region.

### Supplementary information

Supplementary Information

Description of Additional Supplementary Files

Supplementary Movie 1

Supplementary Movie 2

## Data Availability

We have uploaded the source data to the Zenodo database, accessible at: https://zenodo.org/record/7269313. The data supporting the results of this paper are available from the corresponding author upon reasonable request.
