# Supplementary material for: Miniature optical fiber curvature sensor via integration with GaN optoelectronics
Source: Commun Eng. 2022 Dec 27;1:47. doi: 10.1038/s44172-022-00049-w (PMC10955999; doi:10.1038/s44172-022-00049-w)
Supplement: Supplementary file 1 — Supplementary Information [file 44172_2022_49_MOESM1_ESM.pdf]

Supplementary Materials for

Miniature optical fiber curvature sensor via integration with GaN optoelectronics

Fan Shi[1], Hao Zhang[1], Ziqi Ye[1], Xianwu Tang[1], Feifei Qin[1], Jiabin Yan[1,], Xumin Gao[1], Hongbo Zhu[1], Yongjin Wang[1,*], Yuhuai Liu[2] and Hiroshi Amano[3,*]*

* Correspondence to: jbyan@njupt.edu.cn, wangyj@njupt.edu.cn, amano@nuee.nagoya-u.ac.jp

S1. GaN chip fabrication

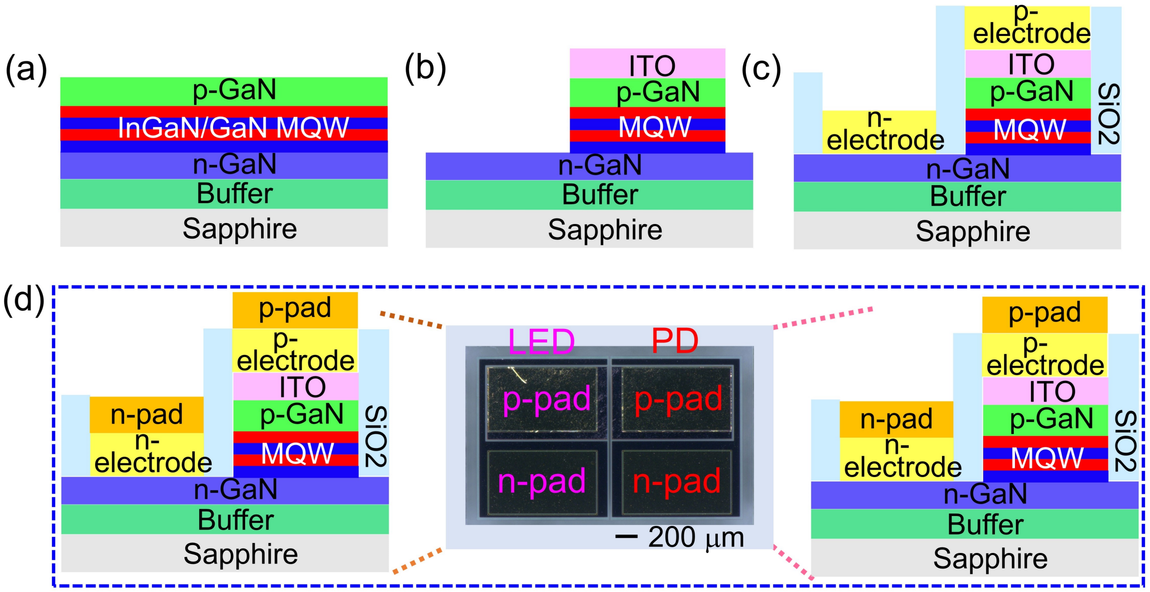

Figure S1. **Schematic diagram of the micro-nano fabrication process.** (a) The initial wafer. (b) ITO deposition and mesa definition. (c) Deposition of a SiO_2 passivation layer for electrical isolation of LED and PD, and deposition of electrodes by e-beam evaporation. (d) The physical image and schematic diagram of the LED-PD chip after deposition of metal pads.

As shown in Fig. S1(a), the initial wafer is a commercial GaN-on-sapphire LED epitaxial wafer, providing the platform for chip integration. First, the same size of 1.2 ×0.72 mm^2 rectangular mesa regions are photolithography defined as the LED and PD regions, and the n-GaN surface is exposed by inductively coupled plasma (ICP) etching, as shown in Fig. S1(b). A 230 nm-thick transparent indium tin oxide (ITO) current spreading layer is deposited on p-GaN, and the unmask ITO is etched away by wet etching. A 1000-nm thick SiO_2 layer is deposited on the wafer by plasma enhanced chemical vapor deposition (PECVD), and apertures are opened on it for the electrodes deposition, as shown in Fig. S1(c). Finally, the metal pads are deposited to form the flip-chip structure, as displayed in Fig. S1(d).

S2. Current Density Characterization of GaN Chip

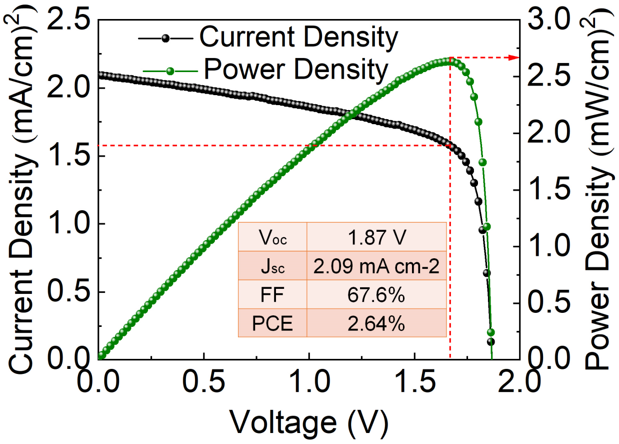

Figure S2. **Current density (black curve) and power density (green curve) of the GaN chip.** Voc, Open-circuit voltage; Jsc, short-circuit density; FF, filling factor; PCE, photon conversion efficiency.

The photoelectric efficiency of the GaN optoelectronic chip is measured to be 2.64% under 1-sun illumination.

S3. Sensing system configuration

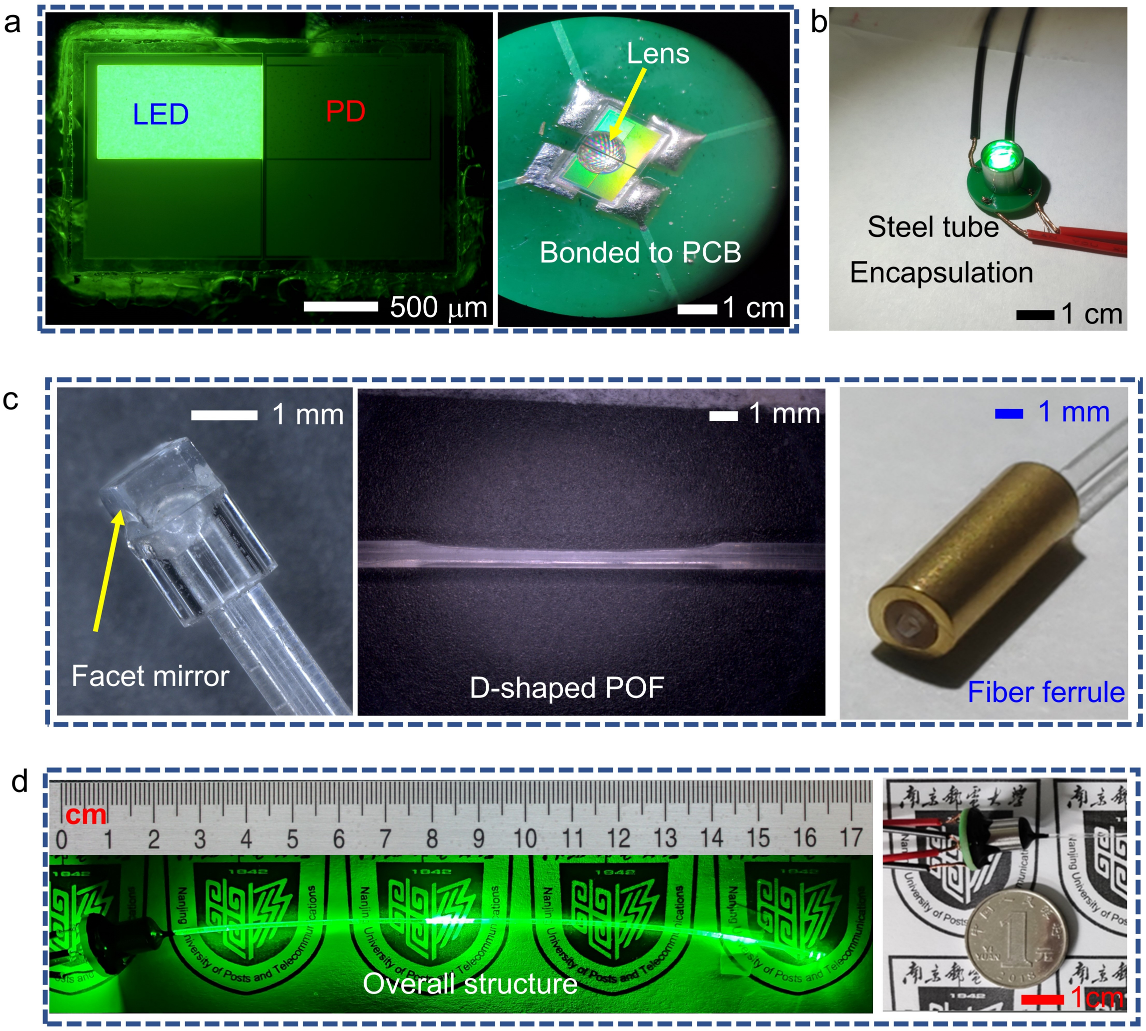

Figure S3. **Fabrication procedure of the POF sensing system.** (a) and (b) Chip and lens packaging with a steel housing. (c) D-shaped POF structure fabrication and facet mirror bonding. (d) Overall structure of the fabricated POF sensing system.

First, the monolithically integrated LED-PD chip is bonded on a print circuit board (PCB) and a drop of high refractive glue is deposited on it as a hemispherical lens. Second, a lens glued into a steel tube is used to focus the light emitted from LED. Then D-shaped structure is fabricated on the middle section of the POF by side-polishing technique, and a mirror is attached to the fiber

facet to construct the reflected sensing channel. The microstructured POF is then fixed with a fiber ferrule. Finally, the overall architecture is formed by combining the packaged steel tube and the fiber ferrule.

S4. Durability test of the sensing system

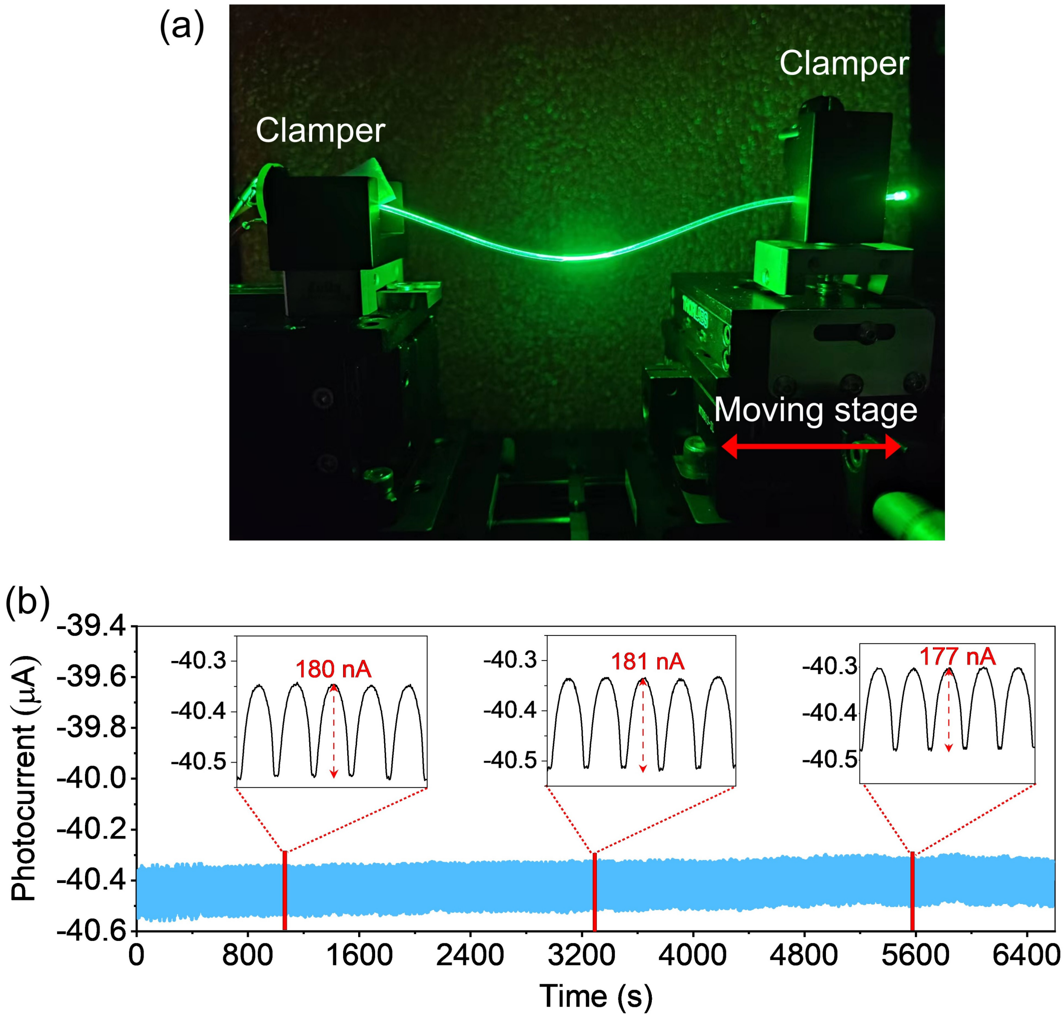

Figure S4. **Durability test of the POF sensing system.** (a) Testing experimental setup. Two clamps are used to hold the fiber optic sensor, and the moving stage moves back and forth to bend and release the fiber. (b) Photocurrent response at curvature of 5.33 m^{-1} is recorded over ~1.8 h. The results indicate that the sensing system is stable and reproducible under high cycles.
