# Supplementary material for: Miniature optical fiber curvature sensor via integration with GaN optoelectronics
Source: Commun Eng. 2022 Dec 27;1:47. doi: 10.1038/s44172-022-00049-w (PMC10955999; doi:10.1038/s44172-022-00049-w)
Supplement: Supplementary file 2 — Description of Additional Supplementary Files [file 44172_2022_49_MOESM2_ESM.pdf]

Description of Additional Supplementary Files

File name: Supplementary Movie 1

Description: Recorded on-chip detection at continuous finger joint bending angles.

File name: Supplementary Movie 2

Description: Recorded on-chip detection at instantaneous finger joint bending frequencies.
